# The SIRT Family: Subcellular Localization and Main Functions

**DOI:** 10.3390/ijms27136042

**Published:** 2026-07-06

**Authors:** Ping Quan, Christoph Schatz, Daria Maria Filippini, Johannes Haybaeck

**Affiliations:** 1 Independent Researcher, 8605 Kapfenberg, Austria; 2 Tyrolpath Obrist Brunhuber GmbH, Hauptplatz 4, 6511 Zams, Austria; 3 Osteoncology, Bone and Soft Tissue Sarcomas and Innovative Therapies, IRCCS Istituto Ortopedico Rizzoli, 40136 Bologna, Italy; 4 Institute of Pathology, Salzkammergut-Klinikum Vöcklabruck, 4840 Vöcklabruck, Austria; 5 Institute of Pathology, Medical University of Maribor, SI-2000 Maribor, Slovenia

**Keywords:** sirtuin (SIRT), sarcoma, expression, prognosis indicator

## Abstract

The mammalian sirtuins (SIRTs), consisting of seven members (SIRT 1–7), are NAD^+^-dependent histone deacetylases (HDACs). Similar to other classical NAD^+^-independent HDACs, SIRTs regulate a wide range of key biological processes by deacetylating both histone and non-histone proteins. By linking cellular metabolism to tissue homeostasis, SIRTs play important roles in physiological regulation and are often deregulated in many human diseases including diabetes, neurodegeneration and cancer, particularly sarcomas. Here, we reviewed the expression and roles of SIRTs, especially the most studied family member SIRT 1, across several types of human sarcomas, including both bone and soft tissues sarcomas. We also discussed the clinical relevance of SIRTs and the potential of their modulation as a therapeutic strategy in sarcomas.

## 1. Introduction

Sirtuins (SIRTs) are an evolutionarily highly conserved protein family homologous to yeast silent information regulator 2 (Sir2), which is characterized as a gene required for maintaining silent chromatin in yeast. The mammalian SIRT family, consisting of seven members (SIRT 1–7), shares a similar ~275 amino acid catalytic domain, as well as similar functions to Sir2. SIRTs are also known as class III histone deacetylases (HDACs), due to their NAD^+^ binding and catalytic domain, which distinguishes them from other NAD^+^-dependent HDACs (classes I, II, and IV) [1]. The seven members of the SIRTs in humans have distinct but not exclusive subcellular localizations and substrates. SIRTs 1, 3, 6, and 7 are primarily localized in the nucleus, whereas SIRT 2 resides exclusively in the cytoplasm, and SIRTs 3–5 are mostly in mitochondria. SIRTs 1–3, and 5 catalyze NAD^+^-dependent deacetylation of targets, whereas SIRTs 4 and 6 have monoribosyltransferase activities. SIRT 5 is an NAD^+^-dependent protein lysine demalonylase and desuccinylase [2,3]. SIRTs 1 and 3 are expressed in a wide variety of tissues and target numerous proteins [4].

Similar to other classical HDACs, SIRTs exert their biological functions by deacetylating both histone and non-histone proteins. A key function of SIRTs is their regulation of transcription repression, mediated through the binding of SIRTs with other proteins [3]. Thereby, SIRTs link cellular metabolism to tissue homeostasis, participating in a wide range of cellular processes with different cellular localizations and molecular targets, e.g., cell proliferation, survival, differentiation, DNA damage and stress response, genome stability, metabolism, and energy homeostasis; additionally, they are involved in organ development, inflammation, protection from neurodegenerative diseases, aging, and organismal longevity, as well as the initiation and development of tumors [1]. The role of SIRTs, particularly SIRT 1, in carcinomas has been extensively investigated, but they are less studied in sarcomas, a rare and heterogeneous group of mesenchymal origin tumors. Here, we review the expression and roles of SIRTs in several types of human sarcomas, as well as the potential application of SIRT modulations in sarcoma treatment.

## 2. Overview of SIRTs in Sarcoma

Several investigations have reported that SIRT family members are involved in sarcoma pathogenesis. An early study from Dickson et al. reported that SIRT 1 was selectively expressed in tumors differentiating along smooth and striated muscle lines. SIRT 1 expression was detected in 76.5% of leiomyosarcoma (LMS, 39/51), 87% of rhabdomyosarcoma (RMS, 20/23), and several benign tumors including angiomyolipoma (100%, 4/4), glomus tumor (100%, 5/5), and leiomyoma (90%, 9/10), but not in other sarcomas, e.g., alveolar soft part sarcoma, clear cell sarcoma, Ewing’s sarcoma (EwS), gastrointestinal stromal sarcoma, osteosarcoma, and pleomorphic sarcoma [5]. Later, more researchers found that *SIRT 1*-positive expression was common in sarcomas and was primarily present in the nuclei [6,7]. Analyzing 104 sarcoma tissues representing 17 subtypes, Kim et al. observed that 71% of patients had strong SIRT 1 expression, particularly in 65% of LMS (13/29), 81% of synovial sarcoma (SS, 13/16), 82% of undifferentiated sarcoma (9/11), and 100% of malignant peripheral nerve sheath tumors (MNPS, 6/6) [7]. Consistently, Berclaz et al. found strong *SIRT 1* staining in 60% of high-risk soft tissue sarcoma (STS, 100/165 representing 6 subtypes) [6]. *SIRT 1* expression varied between different sarcoma subtypes and was mostly expressed in SS (75%) and undifferentiated pleomorphic sarcomas (67%) [6].

Dickson et al. found that *SIRT 1* levels were not associated with tumor grade but had a trend that closely paralleled the desmin levels in LMS, therefore suggesting that *SIRT 1* was a potential marker of myoid differentiation [5]. However, most researchers found that *SIRT 1* expression was associated with advanced clinicopathological parameters, including stage, grade and metastasis, acting as an independent negative prognostic indicator for sarcoma patients [7,8,9]. *SIRT 1* expression was significantly correlated with the expression levels of cyclin D1, ß-catenin, and *P53*, which were evenly correlated with the mitotic count, Ki67 index, advanced stage, distant metastasis, shorter overall survival (OS) and event-free survival (EFS). Strikingly, the *SIRT 1*-positive patients had an 8-fold higher Ki67 index, 10-fold greater risk of death, and 2.5-fold lower EFS [7].

In contrast, in a long-term follow-up of 163 patients with high-risk STS, Berclaz et al. found that the *SIRT 1* expression level was associated with a more favorable prognosis. Patients with high *SIRT 1* levels showed a five-year OS rate of 53% and a median OS of more than 120 months, compared with a five-year OS rate of 35% of and a median OS of 63 months in patients with low *SIRT 1* levels. Moreover, *SIRT 1* expression did not correlate with tumor grading, staging or tumor response but positively correlated with DNA Topoisomerase II Alpha (*TOP2A*). In a multivariate analysis, the high level of *SIRT 1* combined with low levels of *TOP2A* emerged as a specific biomarker of improved survival. Thirty-nine patients (23%) with high *SIRT 1* and low *TOP2A* levels had a 5-year OS of 80% and 10-year OS of 67% vs. 40–50% for 5- and 10-year OS in other patients, respectively. The authors suggested that this disagreement is due to the dual roles of *SIRT 1*. *SIRT 1* might swap from a tumor suppressor to an oncogene after reaching a certain stress threshold [6].

Another analysis of several Cancer Genomics datasets revealed amplification of the *SIRT 7* gene in several types of sarcomas. *SIRT 7* levels were even more dramatically elevated in metastatic tissues compared to the primary tumors [10]. *SIRT 7* is a key regulator of metastatic phenotypes in both epithelial and mesenchymal cancer cells, as it controls cellular invasiveness and extracellular signaling molecules. *SIRT 7* deletion reduced *MMP16* and *VEGF-A* expression, inhibited in vitro migration and invasion, as well as the in vivo metastasis of HT1080 fibrosarcoma cells [10]. *SIRT 7* depletion in HT1080 and U2OS osteosarcoma cells severely impaired both anchorage-independent cellular growth in soft agar and proliferation in low serum conditions. Co-expression of *SIRT 7* and *ELK4*, but not either one alone, synthetically enhanced sarcoma cell phenotypes [11]. Moreover, the interaction of *SIRT 7* with *SIRT 1* is required for the *SIRT 1*-dependent downregulation of *E-cadherin*. *SIRT 1* might function as a scaffold protein in recruiting *SIRT 7* to the E-cadherin promoter and facilitating the *SIRT 7*-mediated deacetylation of *H3K18Ac* [10].

**Figure 1:**
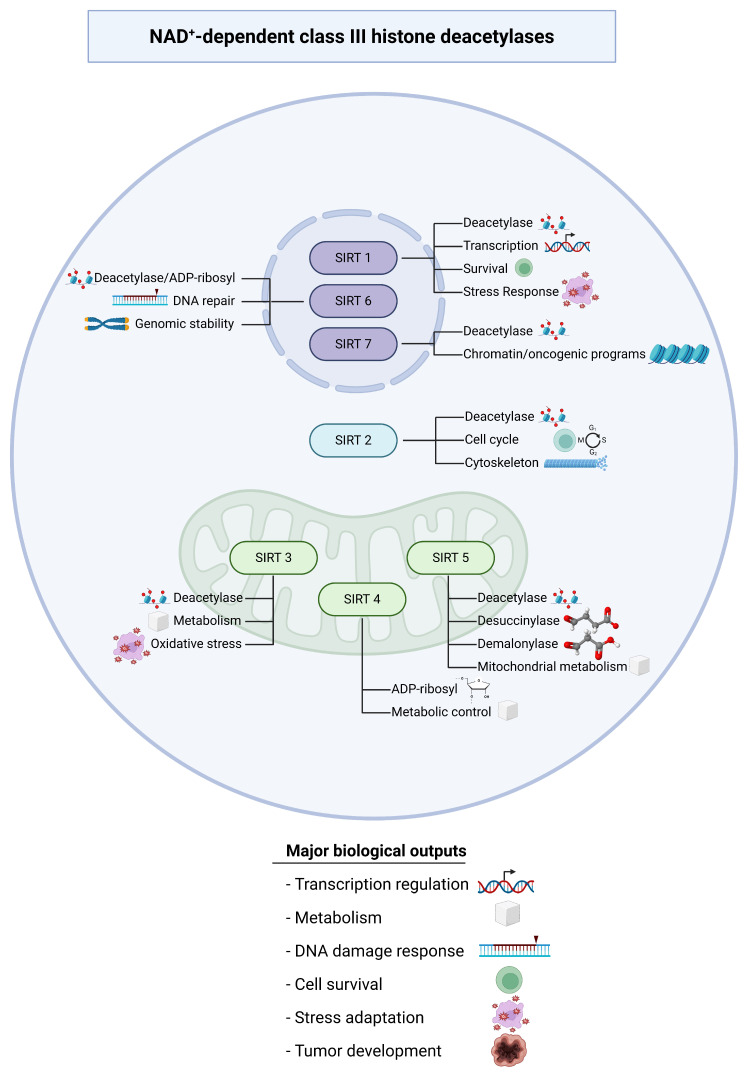
Schematic overview of SIRTs, functions and locations within the cell. Created in BioRender. Science, Y. (2026) https://BioRender.com/a1bcg0s.

In the following sections, we will review the expression patterns and biological roles of SIRTs in several types of human sarcomas (Table 1, Figure 1).

## 3. Altered SIRT Expression and Function in Specific Sarcoma Types

### 3.1. Bone Sarcomas

#### Chondrosarcoma (CHS)

Feng et al. observed *SIRT 1* overexpression in pelvis CHS. Nuclear SIRT 1 protein was positively expressed in 73.5% (25/34) of patients and was associated with tumor size, recurrence, metastasis, pathological type, and poor prognosis. Among patients with positive *SIRT 1* expression, 80% had a tumor size ≥10 cm, 64% developed recurrence, 68% presented with metastases, and 72% had high-grade tumors. By contrast, among patients with negative *SIRT 1* expression, only 22% had tumors ≥10 cm, 11% developed recurrence, 22% had metastasis, and 11% had high-grade tumors [23].

A recent study from Suh et al. reported increased *SIRT 1* expression in 94 CHS samples. Elevated *SIRT 1* expression was particularly associated with high-grade tumors, including grade 3 and dedifferentiated CHS [24]. Gene set enrichment analysis showed that the aggressive phenotype of CHS was linked to upregulated NAD^+^ biosynthesis-related expression profiles. Accordingly, *SIRT 1* silencing could reduce the expression of gene sets associated with CHS malignancy [24,25].

Suh et al. found that *SIRT 1* promotes CHS malignancy and enhances tumor cell survival, wherein *SIRT 1* acts as a central mediator to reinforce the dependency of CHS cells on NAD^+^ metabolism via HIF-2α-mediated transcriptional reprogramming. *SIRT 1* interacts with and deacetylates *HIF-2α*, resulting in enhanced *HIF-2α* expression and activity [24]. SW1353 cells overexpressing *SIRT 1* exhibited a significant increase in colony formation compared to the control group, which was completely abolished by HIF-2α knockdown. *SIRT 1* knockdown reduced CHS cell proliferation and enhanced apoptosis, resulting in effectively suppressed tumor growth and osteolytic expansion, as well as restricted extraosseous outgrowth in vivo in orthotopic xenograft mouse models [24].

The *SIRT 1–HIF-2α* axis is particularly important for the survival of tumor cells under harsh metabolic challenges. It was found that glucose deprivation alone failed to induce the apoptosis of CHS cells because glucose starvation significantly elevated the relative abundance of NAD^+^ to NADH (a deterministic factor for *SIRT 1* activation) and, in turn, stabilized *HIF-2α*. However, *SIRT 1* knockdown or inhibition substantially induced apoptosis in glucose-deprived CHS cells [24].

*SIRT 1* also enhances the migration, invasion and metastasis of CHS via promoting EMT transition. *SIRT 1* overexpression promoted a mesenchymal phenotype, as evidenced by the increased expression of vimentin, N-cadherin and *twist*, together with reduced expression of the epithelial markers E-cadherin and β-catenin. Conversely, *SIRT 1* deletion demonstrated a reversal tendency [23].

However, *SIRT 1* activation by resveratrol inhibited CHS growth and induced cell apoptosis [26,27]. Resveratrol upregulated *SIRT 1* expression and activity, which reduced *p65* acetylation and the activation of *NF*-*κB* [26] and *STAT3* [27], in turn leading to caspase 3 cleavage and *BCL-2* downregulation. These effects were compromised by *SIRT 1* knockdown [26,27] or the HDAC inhibitor MS275 in CHS cells [26] (Table 2).

### 3.2. Ewing Sarcoma (EwS)

SIRT 1 expression was increased in EwS, albeit to varied degrees in different studies. Kim et al. reported positive *SIRT 1* protein expression in 83% (5/6) of EwS patients [7]. Ban et al. observed marked upregulated *SIRT 1* gene expression in 59 primary EwSs as compared with 89 normal tissues [29]. A further analysis of 298 EwS samples showed only 32% (77 cases) with moderate and strong nuclear *SIRT 1* staining and 68% (185 cases) with negative and weak *SIRT 1* staining. However, strong *SIRT 1* expression indicated a high relevance to EwS metastases. Primary tumors associated with lung metastasis or bone metastasis had 50% and 40% strong *SIRT 1* expression, respectively. A detailed analysis of 18 paired EwS samples showed 61% SIRT 1 positivity in primary tumors, 55% in bone metastasis (6/11 cases), and particularly 88% in lung metastasis (7/8 cases). Survival analysis of 43 EwS patients showed that the 5-years OS rate was only 25% in the 12 *SIRT 1*-positive patients, compared with 70% in the 31 *SIRT 1*-negative patients. Of these 43 patients, 19% were *SIRT 1* positive with localized tumors (6/32), and 55% were *SIRT 1* positive with metastatic tumors at diagnosis (6/11) [29]. The high levels of *SIRT 1* were not associated with the death rate but with the metastatic rate. The metastatic rate increased from 0% (0/2) in the slightly strong group to 20% (2/10) in the strong group and to 40% (4/10) in the very strong group. *SIRT 1* knockdown, but not *SIRT 2,* significantly reduced EwS cell viability [29].

### 3.3. Osteosarcoma

The expression and biological roles of SIRTs were extensively investigated in osteosarcoma. Most studies reported that *SIRTs 1*, 6 and 7 were overexpressed in this malignancy and were associated with poor prognosis.

Increased *SIRT 1* expression was consistently observed in approximately 80% of osteosarcoma tissues at both the mRNA and protein levels [14,15]. Feng et al. reported high and moderate *SIRT 1* expression in 55.1% and 24.7%, respectively, of 89 osteosarcoma patients [15]. Zhang et al. found strong *SIRT 1* immunoreactivity in 87.9% of 33 primary osteosarcoma tissues, and only 3% were negative for *SIRT 1* expression. By contrast, adjacent normal tissues showed evident *SIRT 1* expression in only 12.1% of cases, while 54.5% were negative [14]. The gene expression analysis of the Omnibus datasets showed that *SIRT 1* expression was significantly upregulated in high-risk patients [14]. Strikingly, the metastasis samples had the highest *SIRT 1* expression [37].

Nuclear *SIRT 6* expression was found in 49% of 37 osteosarcomas [17]. SIRT 7 genomic amplification was also found in 24% of 149 patients (the TCGA database) and of 58 osteosarcomas (the GEO public microarray data). *SIRT 7* upregulation was evidenced further in another 32 osteosarcoma tissues at the mRNA and protein expression levels by RT-PCR and Western blot analysis [19].

*SIRT 1* expression is an independent prognostic indicator associated with higher Enneking stage, distant metastasis, shorter OS [15,37], and neo-adjuvant chemotherapy [15]. In particular, the expression level of *SIRT 1* was coupled with metastatic risk, acting as a biomarker for a high metastatic rate in osteosarcoma patients [14]. Additionally, *SIRT 1* expression was significantly positively correlated with Leptin, a crucial regulator of energy metabolism [15]. Moreover, for patients who received neo-adjuvant chemotherapy, only SIRT 1 expression was significantly associated with the OS of osteosarcoma patients in multivariate analysis, whereas univariate analysis demonstrated that both Leptin and *SIRT 1* expression levels were significantly associated with the OS of osteosarcoma [15].

Zhang et al. found that high levels of *SIRT 6* were correlated with latent distant metastasis but not with age, sex, tumor size, stage, histological grade, lymph node metastasis, or distant metastasis at diagnosis. *SIRT 6* positivity was observed in 89% of 9 patients with latent distant metastasis, vs. 36% of 28 patients without distance metastasis. Univariable analysis and Kaplan–Meier survival analysis indicated that *SIRT 6* expression was negatively associated with OS or relapse-free survival, acting as an independent prognostic indicator for patients. Specifically, compared with *SIRT 6*-negative patients, *SIRT 6*-positive patients had a range from 4- to 6.9-fold greater risk of tumor relapse or death, especially in patients treated with postoperative adjuvant therapy [17]. Moreover, analysis of TCGA data showed that high expression of *SIRT 7* correlated with poor OS in osteosarcoma [19].

By contrast, Gao et al. found that the *SIRT 6* gene was downregulated in 112 osteosarcomas and 3 cell lines when compared with adjacent tissues or osteoblastic cells [18]. Patients with low *SIRT 6* expression had a higher histological grade, higher Enneking staging and higher metastasis rate. Of the 72 patients with low *SIRT 6* expression, 68% had histological grade III, 61% had stage III, and 64% had metastasis, compared to 38%, 40% and 28% in 40 patients with high *SIRT 6* expression, respectively. The Kaplan–Meier curve showed that higher *SIRT 6* expression correlated with favorable prognosis, i.e., a better overall survival. At 24 months, this difference was major; however, at 36 months, since most patients died, the difference had vanished [18].

*SIRT 1* promotes osteosarcoma cell migration and invasion [14,37]. Primary osteosarcoma cells expressing higher *SIRT 1* levels have a stronger migration ability than those with lower *SIRT 1* levels. Knockdown of *SIRT 1* significantly reduced in vitro migration and invasion and the in vivo lung metastasis of osteosarcoma cells in a mouse xenograft model. Analysis of gene expression showed that the knockdown of *SIRT 1* profoundly inhibited the translation of several downstream pathways, particularly related to cell migration and invasion [14]. *SIRT 1* overexpression increased N-cadherin expression but reduced E-cadherin, leading to increased epithelial mesenchymal transition (EMT) in osteosarcoma cells, wherein *SIRT 1* forms a positive feedback loop with zinc finger E-box binding homeobox 1 (*ZEB1*), a transcription factor, to induce EMT [37].

*SIRT 1* is a downstream target of several tumor suppressors, e.g., miR-217, miR-148a-3p, and miR-204, in osteosarcoma. A corresponding negative association of *SIRT 1* with miR-204 gene expression was observed in osteosarcoma tissues [13]. *miR-148a-3p* targeted ubiquitin-specific protease 22 (*USP22*), thereby inhibiting *USP22* expression. *USP22* affected the biological functions of OS cells by deubiquitinating *SIRT 1* [38]. *miR-217* overexpression reduced *SIRT 1* expression, leading to inhibited cell proliferation, migration, and invasion of osteosarcoma cells [39]. *SIRT 1* impairs liver kinase B1 (*LKB1*) protein stability via reduced miR-204 expression. miR-204 overexpression or *SIRT 1* depletion led to *LKB1* accumulation and growth arrest in osteosarcoma cells [13].

Additionally, *SIRT 1* directly interacts with and phosphorylates histone 3 at threonine 3 (*H3T3*) in osteosarcoma cells. The *SIRT 1–H3T3ph* axis facilitates osteosarcoma cell autophagy through the transcriptional activation of ATG genes under starvation conditions, thereby promoting the initiation and progression of osteosarcoma [32]. *SIRT 1* also plays an active role in the chemoresistance of osteosarcoma cells via enhancing p-gylcoprotein and *mdr1* expression. *SIRT 1* expression was increased at the mRNA and protein levels in chemo-resistant cells and tissue samples from patients with chemotherapy [31].

*SIRT 2* was found to play a crucial role in osteosarcoma metastasis by promoting EMT and inhibiting *Snail* degradation. *SIRT 2* was highly expressed in osteosarcoma MG63 and SaoS2 cells, together with enhanced cell migration and invasion capacity, compared to human normal osteoblasts [33]. *SIRT 2* upregulates the protein levels of the mesenchymal markers N-cadherin and vimentin, as well as the levels of *MMP2* and *MMP9*. *SIRT 2* knockdown reduced osteosarcoma growth, the expression of mesenchymal markers, and metastasis in lung and liver in vivo in a xenograft mouse model. *SIRT 2* interacts with Snail and inhibits its degradation, thereby upgrading the EMT of osteosarcoma cells [33].

*SIRT 6* is negatively correlated with tumor suppressor miR-654-5p expression in osteosarcoma. miR-654-5p overexpression reduced *SIRT 6* expression; sarcoma cell proliferation, migration, and invasion; and in vivo tumor growth [16]. *SIRT 7* plays important roles in the maintenance of osteosarcoma cell malignances due to enhanced EMT transition and reduced *CDC4* expression [19]. *SIRT 7* selectively deacetylates *H3K18ac* [11,19], leading to deceased *H3K18ac* binding to the *CDC4* gene promoter and transcriptional inactivation of *CDC4* expression [19]. A negative correlation of *SIRT 7* expression with *CDC4* has been found in 281 osteosarcoma tissues. *SIRT 7* silencing inhibited osteosarcoma growth, which could be abolished by *CDC4* deletion. *CDC4* targets many proteins for degradation. Its reduction leads to increased expression of aurora-A, cyclin E, *c-Myc*, *EZH2*, and *ENO1* and, finally, to enhanced tumor growth [19].

Moreover, *SIRT 7* prevents osteosarcoma cell senescence. *SIRT 7* is localized at silent ribosomal DNA (rDNA) repeats and maintains rDNA heterochromatin. *SIRT 7*, as a scaffold, is required for the association of *SNF2H* (a component of the nucleolar heterochromatin-silencing complex NoRC) with rDNA sequences. *SIRT 7* silencing unleashed rDNA instability, resulting in the excision and loss of rDNA gene copies and subsequent acute senescence [35].

Unlike other SIRTs, *SIRT 4* is a tumor suppressor in osteosarcoma. Transcriptome sequencing revealed that *SIRT 4* may be regulated by the oncogene tripartite motif containing protein 2 (*TRIM2*) in osteosarcoma. Overexpressed *TRIM2* was correlated with metastasis induction and lower survival rates in osteosarcoma patients. Increased *SIRT 4* expression caused by *TRIM2* deletion reduced the invasion and migration of sarcoma cells [28]. *SIRT 3* is the only oxidative stress-responsive SIRT gene since it was downregulated in cultured U2OS cells treated with H2O2 [40], while its role in osteosarcoma is still unclear.

In contrast to most previous reports, two studies suggested anti-osteosarcoma roles for *SIRT 1* and *SIRT 6*.

Herranz reported that systemic 3-fold upregulation of *SIRT 1* reduced both the incidence and size of malignant carcinomas and sarcomas (mostly osteosarcomas), although it did not affect lymphoma incidence. While *SIRT 1* overexpression had no impact on mouse longevity, it was associated with improved healthy aging phenotypes, including reduced glucose intolerance, osteoporosis, and poor wound healing [41]. In addition, Gao et al. found that *SIRT 6* delays osteosarcoma progression by inhibiting cell proliferation and invasion via reducing N-cadherin expression at both the mRNA and protein levels [18].

## 4. Soft Tissue Sarcoma (STS)

### 4.1. Angiosarcoma

*SIRT 1* staining was strong in angiosarcoma, compared to its weak and diffuse expression in hemangioma (both 10 cases) [9]. Kim et al. observed 100% (5/5) strong *SIRT 1* expression, which was associated with shorter survival of patients. *SIRT 1* overexpression induced invasive proliferation and malignant transformation. *SIRT 1* reduction by siRNA resulted in the downregulation of cell growth, proliferation, migration, and invasion [7]. By contrast, Berclaz et al. found 60% (3/5) strong *SIRT 1* expression in high-risk angiosarcoma, but it was associated with long OS in patients [6].

Moreover, *SIRT 7* mRNA expression was increased in 15 angiosarcoma patients. *SIRT 7* is the downstream target of *miR-340*, and it inversely correlated with *miR-340* expression in angiosarcoma tissues. *SIRT 7* silencing inhibited the proliferation and invasion of ISO-HAS-B angiosarcoma cells [20].

### 4.2. LPS

In LPS tissues, Wu et al. found that nuclear *SIRT 1* staining varied from low to high among 42 cases: low in 21.4% (9/42), moderate in 31.0% (13/42), and high in 47.6% (20/42) of cases [8]. High *SIRT 1* expression predicted a poor prognosis and was associated with high histological grade, distant metastasis, AJCC staging, high *VEGF* expression, and shorter OS [8]. Kim et al. also reported that high *SIRT 1* expression was associated with a short event-free survival and OS of LPS. Paradoxically, *SIRT 1*-positive expression was found in 40% of 10 myxoid and 75% of 4 well-differentiated LPSs but not in more aggressive dedifferentiated LPS (3 case) [7].

Berclaz et al. observed high *SIRT 1* expression in 54% of 35 LPS patients. Different from other studies, they found that high *SIRT 1* expression was associated with a long OS. In particular, high *SIRT 1* and low *TOP2A* levels were two favorable predicators in high-risk STSs [6].

### 4.3. SIRT Expression in Other Sarcomas

About 75–81% of SSs express *SIRT 1* protein [6,7]. Ma et al. found higher levels of *SIRT 1* mRNA expression in 12 SS biopsies and 7 sarcoma cell lines including SS, RMS cells and MPNST cells, in comparison with 4 normal primary mesenchymal cells [21]. *SIRT 2* levels were significantly lower than *SIRT 1* in most samples and had no differences in sarcomas, in comparison with normal controls [21]. However, both *SIRT 1* and *SIRT 2* are crucial for the survival of SS and RMS cells. *SIRT 1* and *SIRT 2* double deletion reduced in vitro and xenograft growth of RMS cells, and loss of *SIRT 1* expression reduced *LC3II* expression [21]. *SIRT 3* is one of the targets of *miR-761* for chemoresistance of SS to multitarget tyrosine kinase inhibitor pazopanib treatment [36].

In uterine sarcoma, mRNA expression levels of *SIRT 1*, *miR-152* and *miR-24* were lower in the serum of 101 patients compared to healthy controls. *miR-152* and *miR-24* expression was linked with disease stage, and the patients with high levels of miR expression had better survival rates. *SIRT 1* could be activated by the mimics of *miR-152* and *miR-24*, which deacetylate *LC3* to promote autophagy in uterine sarcoma cells [22]. However, whether the levels of serum *SIRT 1* really reflect its expression level in uterine sarcoma needs to be clarified.

## 5. Modulation of SIRT 1/2 in Sarcoma

Several *SIRT 1* and *SIRT 2* modulators have been reported to exhibit beneficial antitumor effects in several sarcomas. However, the currently available evidence remains largely preclinical, and no SIRT-targeting strategy has yet entered into the routine management of sarcoma patients.

### 5.1. SIRT Activators Induce Apoptosis in CHS, EwS and Osteosarcoma Cells

The natural polyphenol resveratrol is one of the most potent *SIRT 1* activators, despite the existing controversy [6]. Resveratrol activated the caspase 3 pathway and induced CHS death [26,27]. Similarly, resveratrol could induce apoptosis in all four tested osteosarcoma cells at all doses while exerting only minimal effects on normal osteoblasts. One synthetic SIRT 1 activator, isonicotinamide, also increased osteosarcoma cell apoptosis, but it had no effect on normal osteoblasts [12]. This apparent selectivity toward malignant rather than non-malignant bone cells may be clinically relevant, as it suggests a potentially favorable therapeutic window.

Resveratrol and another synthetic *SIRT 1* activator, SRT1720, both effectively induced EwS cell death, yet SIRT 1720 was more potent. SRT1720 killed ES cells at concentrations that were one to two orders of magnitude lower than resveratrol. The effect of resveratrol was much the same in the three cell lines, while the effect of SRT1720 varied by about a factor of ten. In addition, the effect of SRT1720 followed a much steeper concentration–response relationship than the effect of resveratrol. It was suggested that SRT1720 is substantially more potent than resveratrol, while it has to be individually adjusted according to the treatment response and, thus, might require a more careful medication [42]. Clinically, this variability suggests that the response to SIRT activation may differ substantially across tumors and may ultimately require patient selection or biomarker-based stratification.

Notably, resveratrol and SRT1720 had contradictory effects in combination with chemotherapy in EwS. Resveratrol decreased whereas SRT1720 largely increased the effectiveness of etoposide and vincristine independently of *p53* status in EwS cells. Resveratrol significantly reduced the etoposide- and vincristine-induced *p21* expression. This effect was attributed to the pleiotropic actions of resveratrol, which modulates several cellular pathways, including the stimulation of AMP kinase, as well as the inhibition of mTOR signaling and cAMP phosphodiesterases in EwS [42]. However, resveratrol had a synergic effect with L-asparaginase to induce osteosarcoma cell apoptosis [12]. Therefore, dietary supplementation with resveratrol should be carefully considered for EwS patients undergoing chemotherapy. Accordingly, potential interactions between supportive supplements and systemic therapy should be considered in future translational and clinical studies (Table 3).

### 5.2. SIRT Inhibitors Tenovin-1 and Tenovin-6 Induced Cytotoxic Effects Dependent on Cell Type and p53 Status

Tenovin-1 and its water-soluble analog tenovin-6 were identified as *p53* activators [46], and their effects were *p53* dependent. One study showed that tenovin-1 dose-dependently induced caspase-mediated cell death in p53 wild-type EwS cells (WE-68). However, p53-null EwS cells (SK-N-MC) were even more sensitive to tenovin-1 than *p53* wild-type EwS cells in a bell-shaped pattern, i.e., low concentration of tenovin-1 was much more effective than higher concentrations. Tenovin-1’s effects in *p53*-null cells were involved in gene expression changes of Bcl-2 family members, mitochondrial membrane depolarization, nuclear translocation of apoptosis-inducing factor AIF, ROS formation and DNA damage; all these effects displayed a similar bell-shaped pattern [43].

EwS cells are also highly sensitive to tenovin-6, and their sensitivity to tenovin-6 is dependent on intact *p53* and *SIRT 1* expression levels. The lowest IC50 values were found in the VH64 and TC252 cell lines (with wild-type *p53* and the highest levels of *SIRT 1*), compared to the highest IC50 values in A673 cells (with *p53* deletion and the lowest levels of *SIRT 1*). Tenovin-6 activity corresponded to *p53* activation, i.e., *p53* acetylation and increased *CDKN1A* expression. Reduced TC252 cell viability caused by tenovin-6 could be rescued with pre-deletion of *p53*. Consistently, tenovin-6 significantly reduced in vivo tumor growth and migration of TC252 cells with WT p53 but not A673 cells in a xenograft zebrafish model [29]. These observations support the hypothesis that p53 status and baseline SIRT 1 expression may serve as candidate predictive biomarkers for the response to SIRT inhibition in EwS. Nevertheless, these markers remain exploratory and require validation in patient cohorts.

However, Ma et al. found tenovin-6 effects on SS and RMS cells independent of *p53* status. Tenovin-6 inhibited the in vitro proliferation of four SS cell lines (wt *p53*) and three RMS cell lines (mutant *p53*), as well as the in vivo growth of RMS xenografts. Tenovin-6 reduced sirtuin activity in two SS cell lines and one RMS cell line, but *SIRT 1*, *SIRT 2* and *p53* expression was not affected. Tenovin-6 impaired the autophagic flux and induced apoptosis, showing accumulations of *LC3II* and autophagosomes [21].

From a translational standpoint, these results indicate that the mechanism of action of SIRT inhibitors may vary considerably across sarcoma subtypes, with relevant implications for trial design and patient selection. They also support the need for histotype-specific rather than pan-sarcoma development strategies.

### 5.3. The Synergetic Cytotoxic Effects of the Combination of a SIRT 1 Inhibitor with CHEMOTHERAPY in CHS

EX-527, a specific and potent SIRT 1 inhibitor, alone inhibited the growth of SW1353 and JJ012 CHS cells. However, co-administration of EX527 and doxorubicin inhibited the growth of JJ012 tumors more significantly, resulting in the smallest tumor by size and weight among the different tested groups. Moreover, among three NAD^+^ biosynthesis pathways, SIRT 1 inhibition exhibited the most significant enhancement of doxorubicin-induced apoptosis in the orthotopic mouse model of SW1353 chondrosarcoma. The combined therapy nearly abolished osteolytic tumor expansion within the tibial medullary cavity and invasive outgrowth of tumors into the surrounding muscle tissue. These findings support the efficacy of SIRT 1-targeted therapy in combination with conventional chemotherapy in CHS [24]. This is one of the most relevant observations in the field, because it suggests that SIRT 1 inhibition may be more valuable as a chemosensitizing strategy than as a stand-alone treatment. Given the limited systemic treatment options for advanced CHS, combination approaches of this type may deserve priority in future translational studies.

### 5.4. Other Substances Modulating SIRT Signaling

Melatonin displays anti-osteosarcoma activity via downregulating *SIRT 1* signaling. It strongly reduced osteosarcoma cell viability, adhesion, and migration and glutathione levels, while increasing apoptosis and reactive oxygen species [44]. In addition, 2-ME, a natural derivative of 17β-estradiol, was identified as a potent inhibitor of *SIRT 3* by binding both the canonical and allosteric inhibitor binding sites. It inhibited mitochondrial biogenesis in osteosarcoma cells via the regulation of *PGC-1α*, *COXI*, and *SIRT 3* expression, thereby inducing the nuclear recruitment of neuronal nitric oxide synthase and nitric oxide generation [45]. Recently, among 1271 well-annotated pharmacological compounds, Nagao et al. identified that two cardiac glycosides, namely, proscillaridin A and lanatoside C, significantly induced cell death and inhibited the in vitro and in vivo growth of two ULMS cell lines (SKN with *p53* and *PTEN* mutation and SK-UT-1 cells with *RB1* mutation and *PTEN* deletion or insertion), mediated through the sirtuin signaling pathway. Both drugs suppress uncoupling protein 2 (*UCP2*) expression, a family of mitochondrial anion proteins, leading to increased reactive oxygen species (ROS). *UCP2* was significantly upregulated in ULMS tissues compared with myoma tissues at both the RNA and protein levels [47], indicating a novel therapeutic target in ULMS. Although these findings remain preliminary, they are clinically interesting because they point to the possibility of drug repurposing in rare sarcoma subtypes with limited therapeutic options.

## 6. Conclusions and Future Prospectives

SIRTs, especially *SIRT 1*, *SIRT 6* and SIRT 7, were upregulated and were associated with poor prognosis in most of the discussed sarcomas. SIRT 1, SIRT 6 and SIRT 7 promote sarcoma-genesis, particularly associated with metastasis of bone sarcoma, i.e., CHS, EwS, and osteosarcoma. Therefore, SIRTs could act as novel biomarkers and future sarcoma treatment targets. However, the significance of *SIRT 1* expression and modulation in oncogenesis is controversial due to its dual roles. In the early stages of tumorigenesis, the activation of *SIRT 1* increases genome stability, thereby preventing tumor progression [27,41]. However, in the later stages, *SIRT 1* can promote tumor growth by deactivating *p53*, indicating the usefulness of *SIRT 1* inhibition [14,21]. As a consequence, both sirtuin modulations showed beneficial effects, although they were P53 status and sarcoma type dependent. Therefore, a personalized application of SIRT modulation should be considered in clinical therapy. Currently, due to the limited number of studies on SIRTs in sarcoma, there exist huge gaps between basic studies and translational application in sarcoma therapy. In the future, more studies should focus on the sarcoma-subtype- and context-dependent specific roles of SIRT isoforms. The crosstalk between SIRTs, other epigenetic regulations, the signaling pathways, as well as the sarcoma immune microenvironment will be new research directions. To increase the specificity, SIRT 1/SIRT 2 modulators, i.e., EX527, SRT1720, TV1 and TV6, need to be refined. Specific SIRT modulators are urgently needed. Other modulators, e.g., inhibitors of SIRT 6 and SIRT 7 and SIRT 3 activators, need to be investigated. Meanwhile the toxicity of these modulators must be carefully considered. Additionally, future research should focus on the validation of SIRT expression in larger clinical cohorts, ideally including homogeneous sarcoma subtypes, standardized immunohistochemical or molecular assessments, detailed clinicopathological features, and long-term outcome data.

## Figures and Tables

**Table 1:** SIRT expression in sarcomas.

Sarcoma	SIRT	Cases	Expression	Clinical Relevance	Reference
Bone sarcoma					
Chondrosarcoma (CHS)	*SIRT 1*	34 pelvis CHSs	74% of patients with positive *SIRT 1* expression	*SIRT 1* expression associated with poor prognosis	[1]
		94 CHSs (15 grade I, 24 grade II, 49 grade III, and 5 dedifferentiated)	High *SIRT 1* expression in CHS, highest in grade III and dedifferentiated CHS	High *SIRT 1* expression associated with high-grade tumors	[2]
Ewing‘s sarcoma (EwS)	*SIRT 1*	59 primary EwSs (the Novartis gene expression atlas), and 446 EwSs in 4 tissue microarrays (TMAs)	Frequent and high expression of SIRT 1 in EwS with metastasis, highest in EwS with lung metastasis	High *SIRT 1* expression associated with metastatic rate and adverse prognosis	[3]
		6 EwSs	83% positive expression	High *SIRT 1* expression associated with short overall survival (OS) and low event-free survival (EFS)	[7]
Osteosarcoma	*SIRT 1*	Osteosarcoma cell lines (HOS, U-2OS, Saos-2 and MG-63)	Upregulated *SIRT 1* expression in sarcoma cells	N. a ^1^	[12]
		14 osteosarcomas	67% of sarcomas with high levels of *SIRT 1* mRNA	Negatively associated with miR-204 expression	[13]
		33 primary osteosarcomas	Overexpressed *SIRT 1* in 87.9% of osteosarcomas	*SIRT 1* expression level as a biomarker for a high metastatic risk in osteosarcoma	[14]
		89 osteosarcomas	Moderate and strong *SIRT 1* expression in 25.8% and 50.5% of patients	*SIRT 1* expression associated with the Enneking stage, distant metastasis and neo-adjuvant chemotherapy as an independent negative prognostic indicator for osteosarcoma	[15]
	*SIRT 6*	63 primary osteosarcomas, and 2 osteosarcoma cell lines (MG63 and U2OS).	High *SIRT 6* expression in osteosarcoma tissues and cells	N. a ^1^	[16]
		37 osteosarcomas	Positive *SIRT 6* expression in 49% of osteosarcomas	*SIRT 6* as an independent negative prognostic indicator of OS or EFS of osteosarcoma	[17]
		112 pairs of osteosarcomas; U2OS, MG-63 and Saos-2 osteosarcoma cells	low *SIRT 6* expression in 64% of patients	Low *SIRT 6* expression associated with high histological grade, high Enneking staging and high metastasis rate, as well as negative prognosis	[18]
	*SIRT 7*	149 osteosarcomas (the TCGA); 90 paired tumors; 4 sarcoma cell lines (SJSA-1, Hs755, MG-63, and D-17)	Upregulated *SIRT 7* in 24% of patients at mRNA level and in sarcoma cells at mRNA and protein levels	High *SIRT 7* mRNA expression associated with poor OS	[19]
Soft tissue sarcomas					
Angiosarcoma	*SIRT 1*	5 angiosarcomas	60% with high *SIRT 1* expression	High *SIRT 1* expression associated with long OS; high *SIRT 1* together with low TOP2A level as favorable predicators	[4]
		10 angiosarcomas	Overexpressed *SIRT 1* in angiosarcoma	N. a ^1^	[9]
		5 angiosarcomas	100% positive *SIRT 1* expression	*SIRT 1* expression associated with shorter OS and EFS	[7]
	*SIRT 7*	15 angiosarcomas	Overexpressed *SIRT 7*	*SIRT 7* inversely correlated with *miR-340* expression	[20]
Clear cell sarcoma	*SIRT 1*	1 patient	100% positive expression	*SIRT 1* expression associated with shorter OS and EFS	[7]
Epithelioid sarcoma	*SIRT 1*	4 patients	75% positive expression	*SIRT 1* expression associated with shorter OS and EFS	[7]
Adult fibrosarcoma	*SIRT 1*	5 patients	80% positive expression	*SIRT 1* expression associated with shorter OS and EFS	[7]
Myxofibrosarcoma	*SIRT 1*	4 patients	25% positive expression	*SIRT 1* expression associated with shorter OS and EFS	[7]
Leiomyosarcoma (LMS)	*SIRT 1*	30 LMSs	57% with high expression	High *SIRT 1* expression associated with long OS; favorable predicators: high *SIRT 1* and low *TOP2A* level	[4]
		20 LMSs	65% positive, and primarily nuclear localization of *SIRT 1*	*SIRT 1* expression associated with tumor stage, distant metastasis, histological grade, mitotic count, Ki67 index, and expression levels of cyclin D1, ß-catenin, *p53* and *DBC*1	[7]
Liposarcoma (LPS)	*SIRT 1*	42 LPSs	Low expression in 21.4%, moderate in 31.0%, and high in 47.6% of LPSs	*SIRT 1* positively associated with EGF, as a negative prognosis predicator	[8]
		17 LPSs: 3 dedifferentiated (DD), 10 myxoid, and 4 well-differentiated (WD) LPSs	Positive expression in 40% of myxoid and in 75% of WD LPSs	High *SIRT 1* expression associated with shorter OS, and EFS	[7]
		35 patients	54% with high expression	High SIRT 1 expression associated	[4]
	SIRT 1	9 patients	33% with high expression	with long OS. Favorable predicators: high SIRT 1 and low TOP2A level.	[4]
MPNST	*SIRT 1*	6 patients	100% positive expression	*SIRT 1* expression associated with shorter OS and EFS	[7]
	*SIRT 1* and *SIRT 2*	MPNST cell line 740728	No difference vs. normal cells	N. a ^1^	[21]
Low-grade myofibroblastic sarcoma	*SIRT 1*	2 patients	50% positive expression	*SIRT 1* expression associated with shorter OS and EFS	[7]
Rhabdomyosarcoma (RMS)	*SIRT 1*	3 alveolar ARMSs, 2 embryonal ERMSs, and 2 pleomorphic PRMSs	100% positive in ARMS, 50% positive in ERMS, and 100% positive in PRMS	*SIRT 1* expression associated with shorter OS and EFS	[7]
	*SIRT 1* and *SIRT*	RD, RMS and RH30 RMS cell lines	No difference vs. normal cells	N. a ^1^	[21]
Synovial sarcoma (SS)	*SIRT 1*	20 SSs	75% with high expression	High *SIRT 1* expression associated with long OS; Favorable predicators: high *SIRT 1* and low *TOP2A* level.	[4]
		16 SSs	81% positive expression	*SIRT 1* expression associated with shorter OS and EFS	[7]
	*SIRT 1* and *SIRT 2*	12 SS samples and 3 cell lines (1273-99, Syo1, Bax)	Only *SIRT 1* overexpressed in SS tissues and cells; lower *SIRT 2* levels than *SIRT 1*	N. a ^1^	[21]
Undifferentiated pleomorphic sarcoma	*SIRT 1*	55 patients	67% with high expression	High *SIRT 1* expression associated with long OS. Favorable predicators: high SIRT 1 and low *TOP2A* level	[4]
Undifferentiated sarcoma	*SIRT 1*	11 patients	82% positive expression	*SIRT 1* expression associated with shorter OS and EFS	[7]
Uterine sarcoma	*SIRT 1*	Serum of 101 patients	Decreased	N. a ^1^	[22]

^1^ N. a is defined as Not applicable.

**Table 2:** Roles of SIRTs in sarcomas.

Sarcoma	SIRT	Role	Molecular Mechanism	Reference
Bone sarcoma				
Chondrosarcoma (CHS)	*SIRT 1*	*SIRT 1*, as a tumor suppressor, induces CHS cell apoptosis via increased *NF-κB-p65* deacetylation and caspase 3 cleavage.	Resveratrol increased protein expression and activity of *SIRT 1* and apoptosis in CHS cells. SIRT 1 siRNA reversed resveratrol’s effects on *NF-κB* inhibition via deacetylating *p65*. *SIRT 1* siRNA reduced caspase 3 cleavage induced by resveratrol.	[28]
		*SIRT 1*, as a tumor suppressor, reduces proliferation and induces CHS apoptosis via inhibiting *STAT3* activation.	Resveratrol dose-dependently reduced CHS cell proliferation and viability, accompanied by upregulation of cleaved caspase 3, *SIRT 1* and Bax levels and downregulation of *BCL-2* and *p-STAT3* levels. Suppressed *STAT3* activation caused by resveratrol was abolished by *SIRT 1* siRNA.	[27]
		*SIRT 1* promotes CHS malignancy via the *SIRT 1–HIF-2α* axis.	*SIRT 1* expression levels positively correlated with *HIF-2α* levels in CHS biopsies. GSEA revealed *HIF-2α* target genes negatively enriched in the transcriptome of CHS cells with *SIRT 1* knockdown. In situ PLA and co-IP assays revealed the interaction of *SIRT 1* with *HIF-2α* in CHS cells and orthotopic CHS. In CHS cells, *SIRT 1* overexpression increased *HIF-2α*; vice versa, *SIRT 1* knockdown suppressed the protein level and activity of *HIF-2α*, as well as its target gene expression. *SIRT 1* overexpression increased CHS cell colony formation, while *HIF-2α* knockdown abolished it.	[24]
		Promotes CHS metastasis by inducing EMT transition.	*SIRT 1* overexpression enhanced in vitro migration and invasion and in vivo metastasis due to increased expression of mesenchymal markers (Vimentin, N-cadherin and twist) and reduced expression of epithelial markers (E-cadherin and β-catenin).	[23]
Ewing sarcoma (EwS)	*SIRT 1*	As a downstream effector of *EWS-FIL1* and a downstream target of the NOCH signaling effector *HEY1*, *SIRT 1* deacetylates *P53*, reduces *TP53* expression, and promotes sarcoma survival.	*HEY 1* reduced *SIRT 1* expression, leading to *P53* stabilization and *CDKN1A* induction in EwS cells with wild-type *TP53*. EWS-FLI1 silencing resulted in *HEY1* induction and *SIRT 1* reduction. Luciferase reporter gene assay showed reduced *SIRT 1* promoter activity by *HEY1*. *SIRT 1* knockdown induced *P53* acetylation and activation, same effects as *HEY1* overexpression. *SIRT 1* knockdown, but not *SIRT 2*, induced *P53* acetylation and EwS cell death.	[29]
osteosarcoma	*SIRT 1*	*SIRT 1*, as the downstream effector of NMNAT1 (a key enzyme in nuclear NAD^+^ synthesis), facilitates the survival of cisplatin-treated osteosarcoma cells, therefore contributing to cisplatin resistance.	*NMNAT1* deletion enhanced actinomycin D induced osteosarcoma cell death, partially via increased *p53* acetylation (one main substrate of *SIRT 1*).	[30]
		*SIRT 1* involved in chemoresistance of osteosarcoma.	Increased *SIRT 1* expression in drug-resistant osteosarcoma cells and biopsies from patients after chemotherapy. *SIRT 1* knockdown reversed the resistance phenotype and reduced P-glycoprotein and *mdr1* expression in osteosarcoma cells.	[31]
		*SIRT 1* promotes osteosarcoma autophagy via phosphorylating H3.	IP showed the interaction of *SIRT 1* with H3. *SIRT 1* knockdown reduced H3T3ph, autophagy and ATG protein expression; vice versa, *SIRT 1* overexpression reversed it.	[32]
		*SIRT 1* promotes sarcoma cell growth and *LKB1* protein degradation via *LKB1* deacetylation.	*SIRT 1* inhibition with sirtinol or antisense oligonucleotides induced growth arrest of osteosarcoma cells, accompanied by increased protein expression and acetylation of *LKB*1.	[13]
	*SIRT 2*	*SIRT 2* promotes EMT and sarcoma metastasis via *interacting with* Snail and inhibiting Snail protein degradation.	Osteosarcoma MG63 and Saos-2 cells showed higher levels of *SIRT 2* expression and enhanced proliferation and migration compared with U2OS osteosarcoma cells and control cells. *SIRT 2* knockdown reduced aggressiveness of MG63 and Saos-2 cells, with reduced vimentin, N-cadherin, MMP-2 and MMP-9 expression. In contrast, *SIRT 2* overexpression increased U2OS cell malignancy. *SIRT 2* knockdown reduced in vivo growth and metastasis of MG-63 cells. IP showed the interaction of *SIRT 2* with *Snail*. *SIRT 2* knockdown reduced *Snail* protein expression, and *SITR 2* overexpression increased it. Proteasome inhibitor MG132 rescued *Snail* reduction by *SIRT 2* inhibitor AGK2.	[33]
	*SIRT 4*	*SIRT 4*, a downstream target of oncogene *TRIM2*, suppresses sarcoma metastasis.	*TRIM2* was associated with metastasis induction and low survival.	[28]
			2. *TRIM2* knockdown increased *SIRT 4* expression but reduced MMP expression and EMT transition, thereby suppressing sarcoma metastasis.	[16]
	SIRT 6	*SIRT 6* promotes sarcoma as a downstream target of *miR-654-5p*. *miR-654-5p* reduces *SIRT 6* expression and inhibits osteosarcoma growth.	TargetScan identified *SIRT 6* as a target gene of *miR-654-5p*. Luciferase reporter assay showed the binding of *SIRT 6* to *miR-654-5p*. *SIRT 6* expression, cell proliferation, migration and invasion were promoted by Anti *miR-654-5p* but reduced by *miR-654-5p* overexpression.	[17]
		*SIRT 6* contributes to doxorubicin (DOX) chemoresistance of osteosarcoma cells via activating DNA damage repair.	SIRT 6 overexpression reduced DOX-induced apoptosis in osteosarcoma cells due to activated DNA damage repair pathway (phosphorylated ATM, *ChK2*, and *P53* but reduced *H2AX*).	[18]
		*SIRT 6* inhibits osteosarcoma cell proliferation and invasion via targeting N-cadherin.	*SIRT 6* overexpression inhibited proliferation and migration of osteosarcoma cell, accompanied by reduced N-cadherin expression at mRNA and protein levels. *SIRT* 6 knockdown induced converse effects.	[34]
	*SIRT 7*	*SIRT 7* promotes osteosarcoma cell survival following genomic stress due to attenuated DNA damage, activated SPAK and *p53* response.	*SIRT 7* knockdown induced PARP cleavage and apoptosis in osteosarcoma cells treated with DOX. *SIRT 7* overexpression delayed the onset of premature senescence and conferred resistance with low-dose DOX; it showed apoptosis resistance with high-dose DOX. *SIRT 7* knockdown induced JNK and *p38* activation, while *SIRT 7* overexpression reduced it.	[19]
		*SIRT 7* promotes osteosarcoma growth and metastasis due to reduced *H3K18ac*, leading to the reduced binding of *H3K18ac* to the promoter region of *CDC4* and subsequently to reduced *CDC4* expression and increased EMT transition.	*SIRT 7* deletion reduced proliferation, colony formation, migration and invasion of osteosarcoma cells; vice versa, *SIRT 7* overexpression increased the aggressiveness of sarcoma cells. *SIRT 7* deletion caused less tumor burden in vivo, while *SIRT 7* overexpression promoted tumor growth and metastasis in vivo. Microarray assay showed *SIRT 7* overexpression downregulated CDC4 and its downstream targets; vice versa, *SIRT 7* deletion increased it. TCGA data showed *SIRT 7* negatively correlated with *CDC4*. *SIRT 7* overexpression downregulated *H3K18ac* expression and decreased *H3K18ac* binding to the promoter region of *CDC4*; vice versa, *SIRT 7* deletion increased it. *SIRT 7* deletion increased epithelia mark expression but reduced mesenchymal marker expression.	[35]
		SIRT 7 protects osteosarcoma cells against senescence and acts as a scaffold to stabilize SNF2H protein at rDNA promoters for chromatin silencing.	IP showed the binding of *SIRT 7* to both the *SNF2H* and *TIP5* subunits of *NoRC*. *SIRT 7* overexpression increased *SNF2H* protein expression, and conversely, *SIRT 7* deletion reduced *SNF2H* expression in osteosarcoma cells. *SIRT 7* deletion increased *γ-H2AX* levels at rDNA, which was particularly higher in nucleoli than in the nucleoplasm, together with the increased fragility of rDNA sequences. *SIRT 7* overexpression reduced nucleolar *γ-H2AX* levels.	[11]
		*SIRT 7* maintains the transformation activity of sarcoma cells via *H3K18ac* deacetylation.	*SIRT 7* selectively deacetylates H3K18ac. *SIRT 7* deletion reduced anchorage-independent growth and low-serum proliferation of osteosarcoma cells.	
Soft tissue sarcoma				
Angiosarcoma	*SIRT 1*	*SIRT 1* promotes the malignant transformation of angiosarcoma.	*SIRT 1* siRNA reduced proliferation, migration and invasion of angiosarcoma cells.	[9]
	*SIRT 7*	*SIRT 7*, as a downstream target of *miR-340*, promotes the growth and invasion of angiosarcoma.	*SIRT 7* deletion inhibited the proliferation and invasion of sarcoma cells. *SIRT 7* expression was upregulated in cells with miR-340 inhibition or downregulated by *miR-340* overexpression.	[20]
Fibrosarcoma	*SIRT 7*	*SIRT 7* promotes fibrosarcoma metastasis.	*SIRT 7* knockdown reduced the invasion and migration of sarcoma cells, accompanied by reduced MMP16 and VEGF-A expression.	[10]
Synovial sarcoma (SS)	*SIRT 1*	*SIRT 1* maintains SS cell survival.	*SIRT 1* knockdown reduced SS cell viability.	[21]
	*SIRT 3*	*SIRT 3,* a downstream target of *miR-761,* increases tyrosine inhibitor pazopanib sensitivity.	*miR-761* is upregulated in pazopanib-resistant SS cells. Gene expression analysis identified *SIRT 3* as one of the potential target genes of *miR-761*.	[36]
Uterine sarcoma	SIRT 1	*SIRT 1* induces autophagy via deacetylating LC3 as the downstream effector of *miR-152* and *miR-24*.	Higher expression levels of *miR-152* and *miR-24* are associated with better survival of uterine sarcoma patients. Mimics of *miR-152* and *miR-24* increased the levels of *SIRT 1* and deacetylated LC3 to induce autophagy.	[22]

**Table 3:** SIRT modulation in sarcoma.

Sarcoma	Cells	Drug	Dose	Molecular Effects	P53 Dependent	Reference
Chondrosarcoma (CHS)	JJ012 cells	*SIRT 1* activator: Resveratrol (Res)	Induces cell apoptosis in vitro from 10–200 μM, and inhibits tumor growth in vivo at 100 mg/kg	Res induced caspase 3-dependent apoptosis and *p65* deacetylation, accompanied by *NF-kb* inhibition.	N. a ^1^	[26]
	SW1353 cells	Res	Significantly reduces SW1353 cell viability in vitro from 10–100 μM	Res reduced *Bcl-2* expression and *STAT3* activation but increased *Bax* and caspase 3 expression, which was abolished by *SIRT 1* silencing.	N. a ^1^	[27]
	JJ012, SW1353, and OUMS-27 cells	SIRT 1 inhibitor: EX527	100 μM	1. EX527 induced cell death and reduced colony formation. 2. EX527 with doxorubicin had synergistic cytotoxic effects in vitro and in vivo in a xenograft or orthogenic mouse model. 3. EX52 abolished the glucose-deprivation-induced stabilization of *HIF-2α*, causing CHS cell death.	N. a ^1^	[24]
EwS	WE-68 (wt p53), SK-ES-1 (mutant p53), and SK-N-MC (null p53) cells	*SIRT 1* activator: Res and SRT1720	1. Res induced cell death from 5–100 µM in all cell lines. 2. SRT1720 concentration for equivalent effects in different cells: 8–24 µM (WE-68 cells); 4–10 µM (SK-ES-1 cell); 2–3 µM (SK-N-MC cells).	1. All cell lines were similarly responsive to Res but differentially responsive to SRT1720. 2. SRT1720 was more potent than Res. 3. Res decreased but SRT1720 increased chemotherapy effectiveness (etoposide and vincristine).	Independent on *p53* status of cells, but SRT1720 was more potent for the *p53*-null SK-N-MC cells.	[42]
	SK-N-MC (*p53* null) and WE-68 (wt *p53*) cells	SIRT inhibitor: Tenovin-1 (TV1)	Higher efficiency: 1.4 uM for SK-N-MC cells and 7 uM for WE-68 cells	TV1 dose-dependently reduced cell viability of *p53* wild-type ES cells, while, TV1 at lower concentration was stronger than higher concentrations in *p53*-null ES cells.	Dependent on *p53* status	[43]
	VH64 and Tc252 (with wt *p53*), STA-ET-7.3 (with mutant *p53*), SK-N-MC (truncated *p53*) and A673 (*p53* deletion)	*SIRT 1/2* inhibitor: Tenovilin 6 (TV6)	1. IC_50_ values: between 0.8 and 8.0 μM, with the lowest in VH64 and TC252 with intact *p53* and high *SIRT 1* expression levels; 2. In vivo 6 µM.	TV6 reduced in vivo tumor growth and migration of TC252 cells (with *p53*) but not of A673 cells (without *p53*) in a zebrafish xenotransplantation model.	Dependent on intact *p53* and *SIRT 1* levels	[29]
Osteosarcoma	HOS, Saos-2, U-2 OS, MG-63, and a normal human osteoblast NHOst	*SIRT 1* activator: Res and isonicotinamide	Res at different concentrations: 5, 12.5, 25, 50 and 100 μM; isonicotinamide at 1.25, 2.5 and 10 mM.	1. Res and isonicotinamide induces cell apoptosis at all doses but had minimal effects on normal osteoblasts (except Res 100 μM). 2. l-Asparaginase with Res synergically induced cell apoptosis.	N. a ^1^	[12]
	Human osteosarcoma cell line SOSP-9607	*SIRT 1* inhibition: melatonin	From 250 μM to 1000 μM	Melatonin reduced *SIRT 1* levels and sarcoma cell growth, with increased acetylated *p53*.	N. a ^1^	[44]
	Osteosarcoma 143B cells	*SIRT 3* inhibitor: 2-Methoxyestradiol (2-ME)	10 pM, 100 pM, 1 nM, 10 nM, 100 nM, and 1 μM	2-ME inhibited mitochondrial biogenesis via the regulation of *PGC-1α*, *COXI*, and *SIRT 3*, leading to nuclear recruitment of neuronal nitric oxide synthase and nitric oxide generation.	N. a ^1^	[45]
RMS	RMS, RH30 and RD with mutated *p53*	*SIRT 1/2* inhibitor: TV6	IC_50_ ranging between 1.3 and 5.5 μM at 48 h.	1. TV6 reduced *SIRT* activity but not protein expression. 2. TV6 inhibited cell proliferation and induced *p21* expression in all tested RMS and SS cell lines. 3. TV6 inhibited autophagic flux and induced the accumulation of *LC3II* and autophagy.	Independent on *p53* status but cell-type and time dependent	[21]
Synovial sarcoma (SS)	K-SS1, SYO-1, BAX, and 127399 with wt p53					

^1^ N. a is defined as Not applicable.

## Data Availability

No new data were created or analyzed in this study. Data sharing is not applicable to this article.

## Abbreviations

The following abbreviations are used in this manuscript:

CHS	Chondrosarcoma
EFS	Event-free survival
EwS	Ewing’s sarcoma
HDACs	Histone deacetylases
IHC	Immunohistochemistry
LKB1	Liver kinase B1
LMS	Leiomyosarcoma
LPS	Liposarcoma
MNPS	Malignant peripheral nerve sheath tumor
OS	Overall survival
SIRTs	Sirtuins
RMS	Rhabdomyosarcoma
SS	Synovial sarcoma
TRIM2	Tripartite motif containing protein 2
USP22	Ubiquitin-specific protease 22
